# Impact of Pretreatment Methods on Yield and Composition of Cold-Pressed Black Cumin (*Nigella sativa* L.) Seed Oil

**DOI:** 10.3390/foods14244234

**Published:** 2025-12-09

**Authors:** Valdas Laukagalis, Živilė Tarasevičienė, Mindaugas Visockis, Kiril Kazancev, Eglė Sendžikienė, Anna Kiełtyka-Dadasiewicz, Saulius Šatkauskas, Aurelija Paulauskienė

**Affiliations:** 1 Department of Plant Biology and Food Sciences, Faculty of Agronomy, Agriculture Academy, Vytautas Magnus University, Donelaičio Str. 58, 44248 Kaunas, Lithuania; valdas.laukagalis@vdu.lt (V.L.); aurelija.paulauskiene@vdu.lt (A.P.); 2 Department of Biochemistry, Research Institute of Nature and Technology Sciences, Faculty of Natural Sciences, Vytautas Magnus University, Universiteto Str. 10-506, 53361 Kaunas, Lithuania; mindaugas.visockis@vdu.lt (M.V.); saulius.satkauskas@vdu.lt (S.Š.); 3 Department of Environment and Ecology, Faculty of Forest Sciences and Ecology, Agriculture Academy, Vytautas Magnus University, Donelaičio Str. 58, 44248 Kaunas, Lithuania; kiril.kazancev@vdu.lt (K.K.); egle.sendzikiene@vdu.lt (E.S.); 4 Department of Plant Production Technology and Commodity Science, University of Life Sciences in Lublin, Akademicka 13, 20-950 Lublin, Poland; anna.kieltyka-dadasiewicz@up.lublin.pl

**Keywords:** oil extraction optimization, antioxidants, fatty acids, phenolic content, ultrasonication, pulsed electric fields

## Abstract

This study aimed to evaluate the effects of different pretreatment methods on the yield and chemical composition of *Nigella sativa* L. (i.e., black cumin) cold-pressed oil. Different pretreatment methods used included convection heating, microwave, ultrasound and pulsed electric fields (i.e., PEF); we investigated their effects on key parameters, including oil yield, fatty acid composition, total phenol and flavonoid content, peroxide value, acidity and antioxidant activity. However, no single pretreatment method was found to be better than all others. Instead, ultrasonication and pulsed electric fields (PEF) showed significant advantages. Ultrasonication showed the highest total phenolic content and improved oxidative stability, while PEF improved flavonoid content and antioxidant activity. Therefore, these findings suggest that a combination of several pretreatment options should be considered depending on specific industrial goals related to quality improvement and sustainability. This research also contributes to the existing knowledge on *Nigella sativa* L. oil processing and provides additional insights for optimizing extraction technique.

## 1. Introduction

*Nigella sativa* L., traditionally named black cumin, is an herbal plant broadly recognized for its rich bioactive compounds and remedial properties. Naturally, black cumin seeds and their oil have been long used in various cultures for their attributed health benefits, such as anti-inflammatory, antioxidant, antimicrobial and anticancer properties [1,2]. These attributes are linked to the seeds’ unique chemical composition, which includes essential fatty acids, phenolic compounds, flavonoids and other constituents such as thymoquinone [3]. With increasing demand for functional foods and natural products, black cumin seed oil receives expanded attention from researchers and food industry.

Black cumin seeds, oil and their products, including dietary supplements and functional beverages, are now easily available online and in health food stores globally, hence indicating increasing consumers’ acceptance and demand. These products are usually merchandised for their specific attributes, such as antioxidant and anti-inflammatory properties, thus highlighting the originality and value of *Nigella sativa* L. However, the appointed extraction method and seed preparation can heavily affect the yield and quality of black cumin oil or its related products. Therefore, cold-press extraction is often recognized as an effective method for manufacturing high-quality oils [4]. According to Moghimi et al. (2018), the cold-press method preserves higher concentrations of total phenolic compounds (i.e., TPC) and flavonoids, with reports showing that cold-pressed oil can contain from 20 to 30% more phenolic compounds than solvent-extracted oil [4].

In comparison, solvent extraction method frequently leads to substandard oil yields, especially for seeds with dense or hard cell wall structures such as *Nigella sativa* L., where the average cold-press oil recovery is typically bound to roughly 25 to 27% [5]. On the contrary, supercritical CO_2_ extraction is a feasible substitute method that achieves high extraction efficiency (up to 40%). However, the high cost of the necessary equipment and the complexity of this process often make this method impractical for small producers [6]. Although solvent-based extraction techniques, including hexane extraction, can also achieve higher yields often surpassing 35%, such methods present various deficiencies. In addition to environmental and safety issues, solvent residues may remain in the oil and thus affect its chemical profile. Furthermore, exposure to organic solvents can result in a loss or change of volatile aromatic compounds which negatively impact the oil’s sensory profile [3,6,7,8].

Considering these different extraction methods, cold pressing is a cost-effective, mechanically simple and convenient method for manufacturing clean-label products. To highlight the yield limitations of the cold-pressing method, several non-thermal pretreatment techniques, including ultrasonication, microwave and pulsed electric field (i.e., PEF), have been studied for their potential to improve extraction efficiency and oil quality parameters [4]. Specific physical aspects, such as cavitation (i.e., ultrasonic), rapid internal moisture expansion (i.e., microwave) and electroporation (i.e., PEF), disrupt seed cell structures, thus improving the extraction of oil and bioactive compounds [9,10]. Bakhshabadi et al. (2017) observed approximately a 20% increase in black cumin seed’s oil yield by using microwave pretreatment [11]. Meanwhile Moghimi et al. (2018) reported a significant increase in total phenolic content (i.e., TPC) following ultrasonic application of *Nigella sativa* L. seeds [4,11,12].

Moreover, aligned pretreatment methods affect not only oil’s bioactive compounds but also its fatty acid composition and antioxidant properties. Previous studies have shown that aligned pretreatment methods can change proportions of saturated, monounsaturated and polyunsaturated fatty acids by up to 5% and alter antioxidant activity by over 10%, depending on the pretreatment used [13,14]. These variations highlight the need to systematically evaluate how specific pretreatment methods influence both the nutritional composition and oxidative stability of *Nigella sativa* L. oil.

Nevertheless, there is a growing demand for sustainable food production technologies. Thus, non-thermal methods like ultrasonication and PEF strongly align with present global trends leaning towards energy-efficient and environmentally friendly practices while preserving functional properties and nutritional values of foods. Therefore, such methods are becoming more appealing for industrial applications [15].

While earlier works such as Moghimi et al. (2018) and Bakhshabadi et al. (2017) demonstrated the effects of individual pretreatments like ultrasound or PEF on *Nigella sativa* oil, their studies did not compare multiple methods following identical cold-press extraction conditions, which is a novel approach providing a different angle to the topic [4,11]. Nevertheless gaps remain to understand complex effects of pretreatment methods on *Nigella sativa* L. oil. Only a few studies primarily focus on such techniques as ultrasonication and pulsed electric fields [4,9]. Thus, there is a lack of detailed comparison of the impact of chosen pretreatments on oil yield, fatty acid profiles, and the preservation of bioactive compounds. Moreover, non-thermal methods show promise at the laboratory scale. Thus, their practical relevance for industrial oil production remains underexplored.

To address this gap, this study evaluates the potential impact of four pretreatment methods: convection heating, microwaving, ultrasonication and PEF. The findings’ aim is to aid the comparable assessment of pretreatment techniques, evaluate their effectiveness in improving oil quality and examine their applicability in the food industry. Therefore, this research provides a new contribution towards present knowledge of *Nigella sativa* L. oil’s properties. Outcomes of this research resulted in several pretreatment methods under identical technological conditions. Moreover, we include a comparison of oxidative and bioactive parameters in a single experimental model.

## 2. Materials and Methods

### 2.1. Materials

Seeds of *Nigella sativa* L. were obtained in September of 2022 from a farm in Poland. Black cumin seeds were collected at the stage of full technological maturity (BBCH 89), from a production plantation with an area of 5 ha. The plants were grown on sandy-clay soil with a neutral pH in an integrated farming system. An average sample of 50 kg was obtained for the study, after cleaning.

### 2.2. Determination of Seeds’ Physicochemical Properties

Seed moisture content was determined by drying the samples at 105 °C in a laboratory oven (Memmert Universal Oven, UN 55 Plus, Memmert GmbH, Schwabach, Germany) until a constant weight was achieved [16]. The fat content of the samples was measured using the Soxhlet method [17,18]. For fiber content determination, the Henneberg and Stohman method was used [19]. Ash content was determined by burning the samples in a muffle oven at 550 °C [20]. The protein content was determined by the Kjeldahl method [21]. The results of fat, fiber, ash and protein were converted to dry matter.

### 2.3. Determination of Seed Color

The color profile of *Nigella sativa* L. (black cumin) seeds was determined using a spectrophotometer (ColorFlex EZ Spectrophotometer, Hunter Associates Laboratory Inc., Reston, VA, USA) operating in the CIE Lab* color space. The obtained L*, a*, and b* values were used to evaluate the color profile of the seeds. The lightness (L*) value ranges from 0 (black) to 100 (white), with higher L* values indicating lighter samples. The a* value ranges from negative (green) to positive (red), and the b* value ranges from negative (blue) to positive (yellow).

Before each set of measurements, the spectrophotometer was calibrated using a standard white and black tile [22]. To ensure accuracy, five readings were taken for each sample, and the average values for L*, a*, and b* were calculated. The results were analyzed using the manufacturer’s digital software, “EasyMatch QC” (Hunter Associates Laboratory Inc., version 4.0).

### 2.4. Seed Pretreatment Methods

Seeds underwent various pretreatment methods, including

- Convection heating at 180 °C for 5 min in a laboratory oven (Memmert Universal Oven, UN 55 Plus, Memmert GmbH, Schwabach, Germany) (the chosen temperature and duration were based on previous studies by Farmanov J. (2021) [23] and pilot trials, which showed that higher temperatures or longer heating times resulted in excessive drying of the seed and thus reduced the cold-pressing performance due to poor residual moisture);
- Ultrasonication treatment for 60 min at a power of 90 W and 55 Hz using an ultrasonic water bath at ambient temperature (Argo Lab AU-65, Argo Lab Ltd., London, England) [4];
- Microwave pretreatment at 540 W for 3 min using a commercial microwave oven (the seeds were used without prior hydration, maintaining their moisture content to evaluate the effect of microwave treatment on dry seeds [11,24,25]);
- Pulsed electric field (PEF) pretreatment with an electric field intensity of 2.5 kW (charged voltage) and a pulse length of 99 μs for 45 s performed with an electroporation system (BTX, Rigol, DS1102E, Biochrom Ltd., Cambridge, England) [11].

Pretreatment parameters—including temperature, power, duration, voltage, and pulse width—were selected based on previous studies on *Nigella sativa* L. and related seeds [4,11,23,24,25] and validated through pilot trials. Table 1 summarizes the applied parameters.

Before the application of ultrasonication and PEF pretreatment methods, black cumin seeds were hydrated (i.e., conditioned) to improve the consistency of the treatments and facilitate seed cells’ structure softening for further extraction phases, thus enhancing seed permeation and cavitation processes by improving electrical and acoustic conductivity.

The conditioning (i.e., hydration) process involved mixing the *Nigella sativa* L. seeds with distilled water at a volume ratio of 1:1.5 (seeds to water, respectfully) and the mixture was left to stand at room temperature for 1 h. After conditioning, seeds were drained using a mesh sieve to remove excess water before proceeding with ultrasonic and PEF pretreatment processes. This step was based on experimental trials which indicated that usage of moderate moisture conditions supports ultrasonic and PEF application without leading to excessive seed clumping.

After ultrasonication and PEF pretreatments, the seeds were dried at 50 °C for 120 min to restore their moisture content to the level prior to the hydration step (Table 2). Thus, hydration itself did not have a significant influence on the oil extraction yield or composition and served primarily as an experiment stabilizer, in contrast to thermal pretreatments.

### 2.5. Oil Extraction

Cold-press extraction was performed using a cold press (Oilpressparts, PR-H100/1, Oilpressparts GmbH and Co. KG, Niederkrüchten, Germany) at a room temperature of 24 °C. Control samples were extracted without seed pretreatment. For the other variables, convection heating, microwaving, ultrasonication, and pulsed electric field (PEF) pretreatments for seeds before cold-press extraction were used.

The cold-press chamber temperature was set to remain below 40 °C throughout the extraction process to minimize thermal degradation of the oil. The screw speed was set at 30 rpm and the feed rate was 100 g/min to ensure a consistent flow of material into the press chamber. The seeds were processed in single-pass mode through the cold-press screw and the resulting oil and residual press cake were collected separately. This setup was based on pilot testing to simulate small-scale industrial cold pressing.

The extracted oil was stored in dark and airtight bottles at a temperature of 4 °C until analysis. Samples for oxidative stability testing were used within 24 h, while all other analyses were conducted within 72 h.

### 2.6. Physicochemical Characteristics of Oil

The free fatty acids and acidity of the oil were evaluated according to LST EN ISO 660:1996 [26], while the peroxide value was evaluated according to LST EN ISO 3960:2001 [27].

### 2.7. Oxidative Stability of the Oil

The oxidative stability of the oil was evaluated according to the LST ISO 6886:2016 standard [28] by determining the induction time (h) using a Rancimat instrument (Rancimat 743, Metrohm AG, Herisau, Switzerland) via an accelerated oxidation test. Oil sample mass was 2.5 g, working temperature was +120 °C and air flow was 10 L/h.

### 2.8. Fatty Acids in Oil

Fatty acid composition was analyzed using the following method. Briefly, 10 mg ± 2 mg of the sample was weighed into a vial; we added 500 µL t-butyl methyl ether and 250 µL trimethyl sulfonium hydroxide (i.e., TMSH). The resulting mixture was homogenized using a shaker (IKA, MS3, IKA-Werke GmbH & Co. KG, Staufen, Germany) and 1 μL of the resulting solution was injected into a gas chromatograph (GC/FID) (Perkin Elmer Clarus, 500, PerkinElmer Life and Analytical Sciences, Shelton, CT, USA) using a split/spitless injector. A capillary column (30 m × 0.25 mm × 0.25 μm) was used (Heliflex^®^ AT™-FAME, Alltech Inc., Nicholasville, KY, USA). The carrier gas (hydrogen) pressure was constant at 90 kPa and the separation ratio was 1:100. Chromatograph temperature program: initial analysis temperature of 100 °C, maintained for 2 min after sample injection. The injection was then increased to 240 °C at a rate of 3 °C/min. After reaching 240 °C the temperature was kept for 5 min. Injector temperature was constant at 250 °C. Detector temperature was maintained at 285 °C [29,30].

### 2.9. Total Phenolic Content (TPC) and Total Flavonoid Content (TFC)

For the determination of TPC and TFC in *Nigella sativa* L. seed oil, ethanol was chosen as a less environmentally toxic extraction solvent [30]. A concentration of 75% ethanol was used for both TPC and TFC analyses [31,32].

The Folin–Ciocalteu method was used for TPC analysis, aligning with the protocol described by Singleton et al. (1999) and Singh et al. (2014) [19,33]. A 20 μL sample of the ethanol extract was combined with 500 μL of distilled water and 20 μL of Folin–Ciocalteu reagent. After a 6 min reaction interval, 1 mL of 20% sodium carbonate solution was added to the mixture. The resulting sample-to-solvent ratio was 1:10 (*w*/*v*). An additional reaction time of 30 min was set, and the samples were placed in the dark. The absorbance of the resulting blue samples was measured at 765 nm using a UV–Visible spectrophotometer. A standard calibration curve was established with gallic acid over the concentration range of 0.02–0.10 mg/mL. The resulting correlation coefficient (R^2^ = 0.996) showed strong linearity. The TPC was expressed as milligrams of gallic acid equivalents (GAE). The overall duration of the experiment was 36 min, handled within an ambient temperature of +22 °C.

The aluminum chloride colorimetric method was used for TFC analysis. Briefly, a 1 mL sample of the ethanol (75%) extract was mixed with 10 mL of 10% aluminum chloride solution adding 2 mL of 96% ethanol and 1 mL of 1M sodium acetate. The resulting sample-to-solvent ratio was 1:10 (*w*/*v*). The mixture was incubated in the dark at a room temperature of +22 °C for 40 min, and the absorbance was measured at 415 nm. Quercetin was used as a reference standard, prepared over a concentration range of 0.01–0.40 mg/mL. The calibration curve for quercetin followed a linear relationship with a correlation coefficient (R^2^ = 0.996) indicating a high degree of linearity. TFC was expressed as milligram quercetin equivalents (QE) [34,35].

### 2.10. Determination of Oil DPPH• Radical Scavenging Activity

The antioxidant activity of *Nigella sativa* L. seed oil was evaluated using the DPPH• (2,2-diphenyl-1-picrylhydrazyl) radical scavenging assay. Briefly, 0.3 mL of the ethanol extract was combined with 5 mL of a 0.1 mM DPPH• solution prepared in methanol. The mixture was then vortexed and incubated in the dark at room temperature for 30 min. The decrease in absorbance, which indicates the scavenging of DPPH• radicals, was determined at 517 nm using a UV-Vis spectrophotometer [32,36]. The calculation of the DPPH• radical scavenging activity was based on the following formula:

$$DPPH•Radical\;Scavenging\;Activity,\%=\left(\frac{A_{b}-A_{a}}{A_{b}}\right)\times 100\%$$

*A_a_*—absorbance value of the test sample (t = 30 min);

*A_b_*—blank absorbance value (t = 0 min).

This method is often used to evaluate the antioxidant capacity of oils and extracts, with percentage expressions allowing a clear comparison of radical scavenging abilities among various samples [35].

### 2.11. Oil Volatile Compounds

An electronic nose (The Heracles II, Alpha M.O.S., Toulouse, France), using ultrafast gas chromatography, was set up to study the volatile components of fixed oils according to the method described by Wojtasik-Kalinowska et al. [37]. Briefly, 1 g of oil was placed in 20 mL glass containers, sealed with Teflon-faced silicone rubber closures, and placed in an automated sampler. Each container was subjected to incubation at +50 °C for 10 min with agitation at 500 rpm. The collected headspace gas was then introduced into a gas chromatography system with two columns of different polarity, a non-polar MXT-5 (5% diphenyl) and a semi-polar MXT-1701 (14% cyanopropyl phenyl), both 10 m long and 0.18 mm in internal diameter. The system included two flame ionization detectors (FID). The injected volume was 2500 µL with an injector temperature of +200 °C and a detector temperature of +270 °C. Each injection was performed in triplicate. The method was calibrated with an alkane solution (n-butane to n-hexadecane) to convert retention times into Kovats indices, which facilitates identification of volatiles using the AroChemBase database.

### 2.12. Statistical Methods

The data were analyzed using one-way analysis of variance (ANOVA) to determine the effects of different pretreatment methods on the physicochemical and bioactive properties of *Nigella sativa* L. oil. Post hoc comparisons were performed using Fisher’s least significant difference (LSD) test at a significant level of *p* < 0.05. PCA was performed using the Alpha M.O.S. Heracles II device to evaluate the influence of pre-treatment procedures on the aroma profile of oils extracted from black cumin seeds by cold-press extraction. Statistical analysis was performed using ‘Statistica 12’ software (Statistica; StatSoft, Inc., Tulsa, OK, USA, version 12). Each extraction was carried out in triplicate; results are expressed as mean ± SD (*n* = 3). Correlation analysis was performed to assess the relationships between different variables.

## 3. Results and Discussion

### 3.1. Chemical Content and Color of Seeds

The chemical composition analysis of *Nigella sativa* L. seeds confirms that they are a good source of plant oil, proteins, and fiber (Table 2). It indicates that the press cake remaining after the oil extraction is also nutritionally valuable and can be reintroduced into the food chain.

The seeds resulted in a fat content of 38.54%, which also aligns with Moghimi et al.’s (2018) findings of a high fat content in *Nigella sativa* L. seeds, thus indicating the seeds’ rich lipid profile [4]. Moreover, high fat content is important for the oil extraction process because it affects the quantity of the oil gained.

The determined moisture content was 5.85%, which is within the optimal range for cold-press oil extraction. This is typically between 5% and 7%. Therefore, maintaining this range ensures optimal seed quality and efficient oil yield [13]. This result also closely aligns with data reported by other studies [24,25]. For instance, Bakhshabadi et al. (2017) observed a similar moisture content in untreated black cumin seeds [11].

Furthermore, seeds were found to have 20.62% protein, highlighting their nutritional value. Bakhshabadiet al. (2017) and Ketenoglu et al. (2020) also noted the significant protein content of black cumin seeds [5,11,38]. The protein profile of *Nigella sativa* L. includes essential amino acids such as lysine, arginine, leucine and methionine, as noted by Albakry et al. (2022) and Zaky et al. (2021) [39,40].

Meanwhile, Choudhury (2023) indicated high fiber content in black cumin seed, emphasizing its applicability in dietary profiles [12]. The data of our study also shows that the resulting 18.05% fiber content of *Nigella sativa* L. seeds is beneficial from a nutritional point of view. However, high fiber content of seeds can complicate the oil pressing stages by increasing resistance during pressing, thus reducing oil yield and requiring additional mechanical–physical energy to overcome the fibrous material’s structural barriers. Previous studies, such as those provided by Savoire et al. (2013), have reported that the fibrous matrix in oil seeds can decrease efficiency of oil release, potentially affecting overall extraction yield [13]. This indicates the importance of considering pretreatment methods, like ultrasonication and PEF, which are known to disrupt seed cell walls and increase oil recovery.

The amount of ash in the black cumin seeds was found to be 4.03%, which is similar to the amount reported by Mohammed et al. (2016) [41].

A relatively high level of phenolic compounds was obtained (the TPC of the seeds was 183.33 mg GAE 100 g^−1^), therefore showing strong potential for antioxidant activity, which is consistent with the scientific literature focusing on *Nigella sativa* L. as a rich source of phenolic compounds [42]. For example, Mohammed et al. (2016) reported a TPC of 172.5 mg GAE 100 g^−1^ for black cumin seeds [41]. Meanwhile, the TFC value was 63.51 mg QE 100 g^−1^. This result is similar to Choudhury et al. (2023) who found 57.8 mg QE 100 g^−1^ in methanolic extracts of *Nigella sativa* L. seeds [12]. These outcomes further support the idea of relatively high antioxidant properties in seeds [42].

Nevertheless, color measurements were found to be consistent with findings from other studies, hence indicating that black cumin seeds have a characteristic dark appearance due to their high pigmentation [11]. The major pigments responsible for this color range include melanin and flavonoids. In particular, quercetin and kaempferol result in the dark hue of the seeds and are associated with their antioxidant properties [43].

### 3.2. Physicochemical Properties of Oil

#### 3.2.1. Cold-Pressed Oil Extraction Efficiency

The cold-press extraction efficiency was significantly influenced by the used pretreatment methods (Table 3).

It was detected that seeds subjected to convention heating had statistically significantly lower yield than seeds treated with microwaves, ultrasonication and PEF.

At the same time, the oil yield of seeds which did not have any pretreatment was found to be 25.64%. This baseline efficiency is consistent with the general expectations for cold-press extraction of black cumin seeds. According to Khan et al. (2016) and Ma et al. (2019), oil yields for untreated seeds have been similar [43,44]. Moreover, Al Juhaimi et al. (2018) reported an oil yield of 24.8% from untreated *Nigella sativa* L. seed samples using cold-press extraction, hence further supporting this research’s findings [45]. At the same time, previous scientific studies have suggested that moderate heating can increase oil yield by reducing viscosity and improving oil flow through the breakdown of cell walls [3]. However, this specific effect was not statistically significant in our study when compared to the control sample seeds.

Seeds which were pretreated with microwaves, ultrasonication and PEF had a statistically higher oil yield than untreated seeds or seeds which were subjected to convection heating. Nevertheless, no significant differences were detected between the above-mentioned pretreatment methods for oil yield.

Furthermore, microwaves transfer energy to seeds through molecular interactions. This causes the temperature to rise, resulting in the vaporization of water. This aspect improves porosity and creates pathways for the movement of oil within seeds. Thus, the efficiency of oil extraction improves [46,47]. This aspect is consistent with the findings by Choudhury et al. (2023) and Gaber et al. (2019), who also observed yield increases in *Nigella sativa* L. and canola seeds [12,48]. Therefore, interaction of different factors of microwave pretreatment is important for obtaining the maximum oil yield.

According to Kanchan Suri et al. (2022), *Nigella sativa* L. seeds with an initial moisture content of 6.25% had 4.60% moisture after microwave treatment at 540 W for 5 min, and oil yield reached 37.6% [49]. After the response surface analyses, seeds with 6% moisture were defined as optimal for the microwave pretreatment of pomegranate seeds at 261 W for 102 s [50].

Jianhua Huang et al. (2022) reported that the highest perilla seed oil yield (43.35%) was obtained from microwave-pretreated seeds at 700 W for 6 min [51]. The initial moisture content of the seeds was increased to 9% [51]. However, the most efficient microwave treatment for the hemp seeds was at 540 W for 10 min (26.35%) with no prior moisture treatment [52]. Nevertheless, according to Samira Oubannin (2024) the roasting process using microwaves did not result in any significant changes to the moisture content, which was found to be 3.1 ± 0.1% for the untreated seeds and 3.03 ± 0.1% for the treated seeds after being roasted at 750 W for 15 min [53].

Ultrasonic pretreatment further improved oil extraction, with the cavitation effects disrupting the cell walls of the seeds and allowing for more efficient oil release. This observation is consistent with that of Moghimi et al. (2018) [4], who found that an enhanced ultrasonic power of 90 W and prolonged time of 60 min led to an oil yield of 39.93%. In the case of ultrasonic pretreatment, the results demonstrate that the power of the applied ultrasonic waves had affected the oil yield rather than the pretreatment time (Moghimi et al. (2018) [4]).

An improvement in extraction efficiency was also observed with PEF pretreatment, where electroporation increased cell membrane permeability and facilitated oil release. This result corroborates Bakhshabadi et al. (2017), who reported similar improvements in oil yield due to PEF-induced cell membrane disruption, compared with non-treated samples [5,11]. However, the microwaves were more effective than PEF (Bakhshabadi et al. (2017) [11]). Depending on PEF pretreatment parameters and oil extraction techniques, sunflower oil yield increased by 9.1 and 12.7% [54,55].

According to J. Midhun et al. (2023), the oil production is relatively modest since pulsed electric field pretreatments do not significantly alter the structural properties of oil seeds compared to the other pretreatment methods [47].

The obtained research data indicate that the efficiency of oil extraction was statistically significantly enhanced by 23.40, 24.96 and 30.27%, respectively, when using microwave, ultrasonic and pulsed electric field (PEF) treatments compared to untreated seeds (Table 3). This highlights the importance of considering factors such as technical feasibility, industrial applicability, and differences in chemical composition when selecting the most appropriate pretreatment method for improving cold-pressed oil extraction. Therefore, a comprehensive analysis of both oil yield and chemical composition is required to determine the true role of pretreatment methods.

#### 3.2.2. Oxidative Stability of Oil

Oxidative stability is an important aspect in consideration of the oil’s shelf-life, which is influenced by pretreatment methods, as observed within this research (Table 3). It is also important to consider that identified PV values are of the fresh cold-pressed *Nigella sativa* L. oil. The peroxide value (i.e., PV, indicator of primary oxidation) showed slight variations between the oil samples. The control sample oil had a peroxide value of 2.12 meq/kg, which is consistent with the results reported by Mohammed et al. (2017) [56]. Thus, this indicates relatively low PV values in fresh cold-pressed oil. Furthermore, the ultrasonically treated oil had the lowest peroxide value (1.85 meq/kg), indicating enhanced oxidative stability. This outcome is consistent with the results of Ma et al.’s (2019) research, who reported that ultrasound could improve the oxidative stability of oils [43]. In contrast, oils pretreated with convectional heating, microwaving and PEF showed peroxide values ranging from 2.12 to 2.39 meq/kg, thus showing no significant improvement over untreated seed oil.

During PEF pretreatment, electric fields can promote the electrolysis of water, leading to the formation of reactive oxygen species (i.e., ROS). These ROS may oxidize unsaturated fatty acids before or during oil extraction. In addition, PEF can disrupt cell membranes and release membrane-bound lipids along with pro-oxidant enzymes, which further catalyze lipid oxidation and increase peroxide values. Consequently, this may lead to peroxide values in PEF-pretreated seeds oil that are comparable to those observed in thermally treated oil (convection heating, microwaves) [11,57].

The lower peroxide value of oil from ultrasound-pretreated seeds may be attributed to the mechanical and thermal inactivation of oxidative enzymes, such as peroxidase and lipase, caused by cavitation during ultrasound treatment, the release of oil bioactive compounds and prevention of auto-oxidation [58].

Moreover, oxidative stability, which was measured by the induction period, indicated the impact of the various pretreatment methods. Convection heating resulted in the shortest induction period for *Nigella sativa* L. oil while other pretreatment methods were not statistically significant (Table 3).

The effectiveness of pretreatment methods is reflected in their ability to influence the structural and functional integrity of oil bodies. Ultrasonic pretreatment improves the fragmentation of the membranes surrounding oleosomes (i.e., oil bodies), thereby enhancing oil recovery whilst ensuring the stability of lipids against oxidation [4,59]. This is reflected in ultrasonically treated oils having the lowest peroxide values at a statistically significant level (Table 3). Conversely, PEF disrupts the phospholipid layer via electroporation, facilitating the release of triglycerides and phospholipids while preserving minor bioactive components such as tocopherols, sterols, and phenolic compounds [11,60].

Microwave pretreatment causes the internal water content of oil bodies to vaporize, resulting in their rupture and improved oil release. However, this process can destabilize membrane proteins, potentially exposing lipids to oxidative damage. Similarly, heating can accelerate the oxidation and degradation of phenolic compounds, as noted by Khan et al. (2016), resulting in lower total phenolic content (TPC) levels [44]. In contrast, non-thermal methods, particularly ultrasonic treatment, have the advantage of preserving the structural integrity of bioactive compounds.

### 3.3. Polyphenolic Compounds of Oil

*Nigella sativa* L. oil contains minor bioactive compounds, including phenolic acids, flavonoids and tocopherols, which contribute to its health benefits. These compounds are vital in determining the oil’s nutritional value and oxidative stability. The extraction efficiency and stability of these compounds are affected by pretreatment methods.

Ultrasonication brings cavitation, where the burst of microbubbles generates shock waves. These waves mechanically disrupt seeds’ cell walls, hence increasing cell permeability and improving oil release without notable thermal damage [58]. Pulsed electric field (PEF) pretreatment works by enabling electroporation processes. This creates nanopores in cell membranes that improve oil diffusion through better membrane permeability [61]. Microwave pretreatment heats internal seed moisture, thus causing vaporization that forms microcracks and ruptures structures of the cell. This increases oil release at the cost of potentially causing protein denaturation and lipid oxidation [62]. Convection heating itself softens seed tissues by uniform thermal exposure. This helps to loosen cell matrices to improve oil flow at the potential cost of accelerated oxidation [63].

Therefore, within results of this research, it is noticeable that the disruption caused by ultrasonication increased the availability of phenolic and flavonoid compounds. This is evident by the higher total phenolic content (TPC) of ultrasonically treated oil samples (Table 4).

Ultrasonic pretreatment resulted in the highest TPC of 87.18 mg GAE 100 g^−1^, showing statistically significant differences compared to other methods (*p* < 0.05), while PEF pretreatment exhibited the lowest TPC value of 69.17 mg GAE 100 g^−1^. This variation can be attributed to the cavitation effect of ultrasonic treatment, which disrupts cell walls and facilitates the release of phenolic compounds into the oil [4,12]. According to Moghimi et al. (2018), increasing the ultrasonic power from 30 to 90 W increased the amount of phenolic compounds from 93.21 to 106.6 ppm [4].

However, the lower TPC observed with PEF pretreatment may result from the degradation of phenolic compounds under the influence of electric fields, as noted in previous studies [42]. These findings align with Choudhury et al. (2023) and Mohammed et al. (2016), who reported variations in phenolic content based on the intensity and type of pretreatment applied [11,41,42]. Thus, ultrasonic pretreatment appears as the viable method for enhancing TPC while PEF requires optimization to mitigate the loss of phenolic compounds. According to Guderjan (2007), the polyphenol content of the oils increased by the strength of PEF treatment [64,65].

No statistically significant differences in TPC were observed between the oil from untreated seeds and the oil from seeds subjected to convection heating (Table 4). The total flavonoid content of the untreated seed oil was 9.74 mg QE 100 g^−1^, whereas the content of the treated seed oil fluctuated between 10.01 and 10.39 mg QE 100 g^−1^.

PEF pretreatment method showed the highest TFC amount with significant differences compared to the control sample, thus indicating its role in improving flavonoid content. The significant increase in TFC for PEF pretreated samples highlights the influence of electroporation in smooth migration of water-soluble flavonoid compounds into the oil base [12,42].

On the other hand, no significant differences were observed in the TFC of oil from seeds which were conventionally heated, pretreated with microwaves or ultrasonicated. Meanwhile, Moghimi (2018) noted that ultrasonic pretreatment increases cell membrane permeability, providing greater flavonoid release into the oil [4].

The control oil sample exhibited a scavenging activity of 19.75% (Table 4), whereas the pretreated oils ranged from 18.01% (ultrasonic pretreatment) to 21.72% (PEF pretreatment). PEF-treated oil exhibited the highest DPPH• scavenging activity, demonstrating a significant difference compared to ultrasonic-treated oil, which exhibited the lowest antioxidant activity. Although ultrasound pretreatment resulted in a higher total phenolic content, PEF-pretreated seed oil exhibited stronger DPPH• radical scavenging activity. Cavitation during ultrasound disrupts cell walls more extensively, releasing both free and bound phenolics, some of which may have lower radical scavenging activity [66]. This suggests that PEF preferentially releases or preserves phenolic compounds with higher antioxidant potential, whereas ultrasound increases the total phenolic content, including compounds with lower activity. Additionally, antioxidant activity depends not only on the quantity of bioactive compounds but also on interactions among phenolics, tocopherols, and other minor components. Therefore, HPLC analysis may be employed to qualify and quantify the individual compounds [67].

Correlation analysis revealed a weak positive correlation with TFC (*r*^2^ = 0.34), TPC and DPPH• activity (*r*^2^ = 0.38). These results indicate that increases in extraction yield and certain bioactive compounds do not always directly mean higher antioxidant activity, reflecting the complex interactions between extraction efficiency and oil composition.

Considering the correlations discussed, research findings demonstrate that pretreatment methods significantly influence the chemical composition, physicochemical properties and oxidative stability of *Nigella sativa* L. oil. Ultrasonic and pulsed electric field (PEF) pretreatments have been shown to be effective methods of enhancing the release of bioactive compounds while maintaining oxidative stability, making them promising techniques for industrial food applications.

### 3.4. Composition of Fatty Acids in the Oil

The composition of fatty acids in black cumin oil changed based on the pretreatment method used. It was noted that significant differences appeared only for specific fatty acids (Table 5). These results also align with other researchers who suggested that choosing the correct pretreatment method can change the fatty acid composition [44,68].

Evaluation of an oil sample without any used pretreatments revealed that the dominating fatty acids in *Nigella sativa* L. oil are palmitic (C16:0), stearic (C18:0), oleic (C18:1), linoleic (C18:2), eicosadienoic (C20:2) and linolenic (C18:3) acids. Detected concentrations of the control sample were as follows: 11.12% palmitic, 2.88% stearic, 23.28% oleic, 54.74% linoleic, 2.32% eicosadienoic and 0.77% linolenic acids. In overall, linolenic acid content in the control sample was significantly higher (*p* < 0.05) when comparing it to the pretreated samples, thus indicating higher vulnerability of omega-3 fatty acids in pretreatment processes. In parallel, the proportions of oleic, linoleic and eicosadienoic acids remained statistically insignificant across all different pretreatments. Meanwhile, the indicated high levels of linoleic acid (an omega-6 fatty acid) and oleic acid (a monounsaturated fatty acid) show that the black cumin oil is of high quality and has potential health benefits [63]. Overall, the fatty acid composition found in this study is in line with the results of other research like Borchani et al. (2010), Ma et al. (2019), Ramadan et al. (2002) and Zhang et al. (2020) [42,43,68,69].

Convectional heating of the seeds before the cold-press extraction process resulted in minor changes in fatty acid composition. However, linolenic acid (C18:3) content decreased significantly compared to the control sample (*p* < 0.05), thus indicating a thermal sensitivity for omega-3 fatty acids. Despite this, other major fatty acids such as linoleic (C18:2), oleic (C18:1) and palmitic (C16:0) remained unchanged, showing thermal stability of the fatty acid profile in average heating conditions. In contrast, Khan et al. (2016) also observed that thermal pretreatment caused only slight changes in fatty acid profiles that were not statistically significant [44,45]. This aligns with an idea that the heating process preserves the stability of the fatty acid profile, resulting only in minimal changes compared to other pretreatment methods.

In contrast, the use of microwave pretreatment caused statistically significant changes in the fatty acid profile. Compared to the control sample, the content of linolenic acid (C18:3) was significantly reduced (*p* < 0.05), while linoleic acid (C18:2) increased. Such results suggest that microwave pretreatment may improve the release of certain polyunsaturated fatty acids while degrading more oxidation-sensitive ones, like linolenic acid. Al Juhaimi et al. (2018) also noticed that rapid heating during microwave pretreatment can cause shifts in fatty acid profiles due to localized overheating [70].

Ultrasonic pretreatment revealed fewer statistically significant differences in fatty acid content than microwave pretreatment. The oil had a lower content of linolenic acid (C18:3) and a higher content of eicosadienoic acid (C20:2) content. Acoustic cavitation, which is caused by ultrasonic treatment, disrupts cell structures and releases lipids without significantly altering the fatty acid profile. Moghimi et al. (2018) and Talebi et al. (2016) also emphasized the importance of ultrasonic treatment in improving the efficiency of lipid extraction while preserving the integrity of fatty acids [4,71].

The use of PEF pretreatment showed a statistical significance regarding an increase in eicosadienoic acid (C20:2) and decrease in linolenic acid (C18:3) compared to the control oil sample (*p* < 0.05). In contrast, amounts of saturated fatty acids did not increase, thus indicating that the decrease in unsaturated fatty acids under PEF conditions did not result in a change toward higher saturation. This shows that PEF influences specific polyunsaturated fatty acids without changing the balance of fatty acid groups.

The polyunsaturated fatty acids accounted for the highest percentage of the total fatty acid content, followed by the monounsaturated and saturated fatty acids. The proportions of these fatty acid groups are very similar to those reported by K. Suri et al. (2022) for SFAs, MUFAs and PUFAs, at 15.10%, 24.58% and 60.21% respectively [49].

Breaking down the fatty acid categories, the saturated fatty acid (SFA) content remained relatively stable across all of the pretreatments (Table 6). There were no statistically significant differences (*p* > 0.05) compared to the control sample.

Choudhury et al. (2023) also observed this thermal and oxidative robustness of SFAs [11]. The monounsaturated fatty acid (MUFA) content showed minimal changes across the pretreatment methods. However, these changes were not statistically significant, indicating that these compounds were not substantially affected. Moghimi et al. (2018) and Oubannin et al. (2022) also reported consistency in MUFA levels across various pretreatment techniques in *Nigella sativa* L. seed oil [4,72]. The amount of PUFA in the untreated and treated seed oils also remained stable. Therefore, the results show that the selection of pretreatment has no significant effect on the relative distribution of PUFA profiles. However, according to Suri et al. (2022) the proportion of MUFAs and PUFAs decreased while the proportion of SFAs increased when *Nigella sativa* L. seeds were roasted at 720 W for 10 min [49].

The research results show that using different pretreatments on the seeds does not significantly affect the fatty acid profile.

However, the levels of omega-3 and omega-6 fatty acids, which are key components of PUFAs, varied significantly depending on the pretreatment method used. The omega-3 content was highest in the untreated and convection heated seeds, while the omega-6 content was highest in the seed oil treated with microwaves, ultrasound, and pulsed electric fields. This suggests that pretreatment methods can alter the ratio of these essential fatty acids.

Research by Djuricic (2021), Balić (2020) and Dabbour et al. (2014) emphasized the importance of maintaining an appropriate omega-6 to omega-3 ratio for achieving optimal health outcomes [73,74,75]. In a typical Western diet, the ratio of linoleic acid to α-linolenic acid ranges from 5:1 to 15:1 [76].

The ratio of polyunsaturated to saturated fatty acids (PUFA/SFA) fluctuated from 3.22 for conventionally heated seed oil to 3.63 for oil from seeds subjected to microwave pretreatment. Meanwhile, pulsed electric field (PEF) pretreatment resulted in a higher PUFA/SFA ratio, showing a significant difference compared to the control samples (*p* < 0.05). These variations remained within a narrow range, showing that non-thermal methods preserved a typical lipid profile while slightly enhancing the unsaturated fraction.

The ratio of unsaturated to saturated fatty acids (U/S) ranged from 1.33 to 1.45. The MUFA/PUFA ratio remained stable across all pretreatments, with the exception of oil extracted from untreated seeds.

Moreover, the fatty acid profile of *Nigella sativa* L. seed oil was not significantly influenced by the pretreatment methods used, as there were no statistically significant differences in the fatty acid groups. Only the amount of omega-3 and omega-6 fatty acids was affected by seed pretreatment. The pretreatment methods used preserved the integrity of the beneficial fatty acids, offering potential advantages for industrial applications.

### 3.5. Volatile Aromatic Compounds of Nigella sativa L. Oil

**Figure 1:**
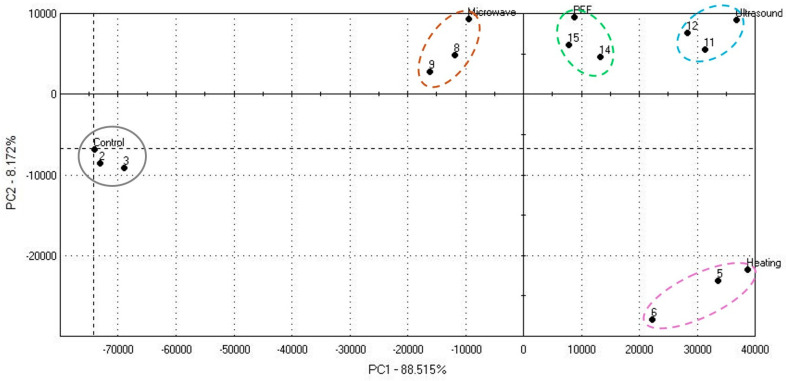
PCA for organic volatile substances in black cumin seeds cold-pressed oils having ungergone different pretreatment methods.

Effects of used seed pretreatment methods on the volatile compound profile for *Nigella sativa* L. oil was evaluated based on the differences in compound presence across different treatments. Principal component analysis (PCA) revealed that used pretreatment methods led to changes in the aroma profile of the cold-pressed oil (Figure 1). The first two principal components (PC1 and PC2) consider 96.69% of the total variance, with PC1 clarifying 88.51% and PC2 8.18%. The PCA score plot showed clear separation between samples based on used treatment type. In this case, non-thermal methods (ultrasonic and PEF) clustering separately from thermal treatment methods (convection heating and microwaving), indicating that pretreatments affect the release or changes of volatile compounds.

Data set explanation of Figure 1:

- Data points of control samples are reflected as group “Control, 2, 3”;
- Data points of samples pre-treated with convection heating are reflected as group “Heating, 5, 6”;
- Data points of samples pre-treated with microwaves are reflected as group “Microwave, 9, 8”;
- Data points of ultrasonically pre-treated samples are reflected as group “Ultrasonic, 11, 12”;
- Data points of samples pre-treated with pulsed electric fields (PED) reflected as group “PEF, 14, 15”.

In total, 12 volatile compounds were identified across the analyzed variations (Table 7). Several volatiles, such as 2-methylpropanal, 2-propanol, dimethyl sulfide and propanal, were detected under the majority of pretreatment methods, while others appeared specifically. In particular, (E,E)-2,4-nonadienal, citronellol and ethyl phenylacetate were found only in ultrasonication-treated samples and carvone was detected specifically only in the PEF-treated sample.

Although some of these compounds have been previously reported in other studies on cold-pressed or refined oils (rapeseed, sesame and soybean oils), their presence in *Nigella sativa* L. oil is not well documented [77,78,79]. In contrast, (E,E)-2,4-nonadienal and carvone have been identified in heat-treated rapeseed oil and roasted sesame oil [80,81]. Similarly, compounds like dimethyl sulfide and 2-methylpropanal have been detected in heat-treated processed edible oils and are often associated with thermal degradation [82,83].

As per these results, (E,E)-2,4-nonadienal, citronellol and ethyl phenylacetate were specifically evident in ultrasonicated samples. Thus, it implies slight oxidation of unsaturated lipids. These compounds can contribute to aroma complexity at low levels but also serve as early indicators of oxidation [84].

Among the other volatiles detected (Table 7), carvone and nerol highlight pleasant herbal and citrus notes typical of high-quality oils [85]. Therefore, while some new volatiles indicated mild oxidation, most of them reflected that non-thermal methods improved aroma without compromising oil quality.

In overall, results suggest that pretreatment methods (specifically non-thermal methods like ultrasonication and PEF) can influence volatile compounds and potentially improve aromatic complexity of cold-pressed black cumin oil. Nonetheless, further studies involving gas chromatography with mass spectrometry confirmation and including sensory evaluation are necessary to further validate the identity and sensory contribution of the volatiles observed in this research.

## 4. Conclusions

This study systematically evaluated the effects of four different seed pretreatment methods (i.e., convection heating, microwave, ultrasonic and pulsed electric field (PEF)) on the yield and quality of *Nigella sativa* L. seed oil.

The findings indicated new insights into how these pretreatment methods may influence the composition and potential quality of cold-pressed black cumin oil. The use of ultrasonication pretreatment significantly increased TPC by 4.77% (*p* < 0.05) and improved oxidative stability, as indicated by the lowest PV value. In contrast, PEF pretreatment improved TFC and antioxidant activity by up to 6.67% and 9.97%, respectively (*p* < 0.05), despite reduced TPC value compared to the ultrasonication method. This discrepancy potentially arises from the differential preservation or release of other antioxidant compounds not analyzed in this study, which contribute to the overall antioxidant capacity. Meanwhile, the use of microwave pretreatment increased oil yield by 6% (*p* < 0.05) but in parallel degraded heat-sensitive components (e.g., TPC value decrease of 0.83% (*p* < 0.05).

These findings imply that non-thermal processing is an energy-efficient alternative to traditional convection heating that can be used on a large scale and for a long time. Moreover, ultrasonication and PEF together may be a promising industrialized technology that increases extraction yield while also protecting sensitive compounds.

Consecutive further studies should focus on assessing integrated methodology in pilot-scale systems, investigating sensory acceptance and storage stability, and conducting economical evaluations to facilitate its implementation in oil production sites.

Overall results contribute to a broader understanding of how pretreatment methods influence cold-pressed oil quality, offering practical applications for optimizing oil production processes.

Ultrasonication and PEF pretreatments represent promising technologies to produce *Nigella sativa* L. oil, offering improvements in phenolic content, antioxidant capacity and compositional diversity.

## Figures and Tables

**Table 1:** Parameters of the pretreatment methods.

Pretreatment Method	Parameters	Expected Effect
Convection heating	180 °C, 5 min	Softening seed texture, mild yield increase
Microwave	540 W, 3 min	Microcrack formation, improved oil diffusion
Ultrasonication	90 W, 55 Hz, 60 min	Seed cell wall disruption, higher oil yield
Pulsed electric fields (PEF)	2.5 kV, 99 μs pulses, 45 s, 30 pulse number, sample temperature +22 °C	Electroporation, higher oil yield

**Table 2:** Chemical content and color of *Nigella sativa* L. seeds. Values are means ± SD (*n* = 3), *p* < 0.05, Fisher’s LSD test.

Parameter	Value
Moisture content, %	5.85 ± 0.02
Fat, %	38.54 ± 0.60
Fiber, %	18.05 ± 0.56
Ash, %	4.03 ± 0.03
Protein, %	20.62 ± 0.54
Color coordinate L*	11.76 ± 0.31
Color coordinate a*	0.12 ± 0.06
Color coordinate b*	−0.49 ± 0.07
Total phenolic content (TPC), mg GAE 100 g^−1^ dry matter	183.33 ± 0.62
Total flavonoid content (TFC), mg QE 100 g^−1^ dry matter	63.51 ± 0.49

**Table 3:** Physicochemical properties of the cold-pressed *Nigella sativa* L. oil. Values are means ± SD (*n* = 3). Lowercase letters “a, b, c” indicate significant differences (*p* < 0.05, Fisher’s LSD test).

Parameter	Seed Pretreatment Methods				
	Control	Convection Heating	Microwave	Ultrasonication	Pulsed Electric Field (PEF)
Oil yield, %	25.64 ± 1.14 ^a^	27.01 ± 1.62 ^a^	31.64 ± 1.47 ^b^	32.04 ± 1.13 ^b^	33.40 ± 1.17 ^b^
Peroxide value, meq/kg	2.12 ± 0.23 ^b^	2.39 ± 0.01 ^c^	2.39 ± 0.02 ^c^	1.85 ± 0.22 ^a^	2.39 ± 0.01 ^c^
Acid value (mg KOH/g oil)	1.99 ± 0.09 ^a^	2.12 ± 0.47 ^a^	1.88 ± 0.15 ^a^	2.11 ± 0.10 ^a^	2.28 ± 0.20 ^a^
Acidity (% of oleic acid)	1.40 ± 0.06 ^a^	1.50 ± 0.33 ^a^	1.33 ± 0.10 ^a^	1.49 ± 0.07 ^a^	1.61 ± 0.14 ^a^
Induction period, h	2.26 ± 0.35 ^b^	1.48 ± 0.20 ^a^	2.02 ± 0.13 ^b^	2.05 ± 0.45 ^b^	1.96 ± 0.12 ^ab^

**Table 4:** Polyphenolic compounds in *Nigella sativa* L. oil. Values are means ± SD (*n* = 3). Lowercase letters “a, b, c, d” indicate significant differences (*p* < 0.05, Fisher’s LSD test). Treatments sharing the same letter (e.g., “a” and “b”) indicate no statistically significant difference (*p* > 0.05, Fisher’s LSD test).

Parameter	Seed Pretreatment Methods				
	Control	Convection Heating	Microwaves	Ultrasonication	Pulsed Electric Field (PEF)
Total phenolic content (TPC), mg GAE 100 g^−1^	83.21 ± 0.11 ^c^	83.23 ± 0.24 ^c^	82.52 ± 0.14 ^b^	87.18 ± 0.23 ^d^	69.17 ± 0.15 ^a^
Total flavonoid content (TFC), mg QE 100 g^−1^	9.74 ± 0.22 ^a^	10.22 ± 0.10 ^bc^	10.01 ± 0.13 ^ab^	10.13 ± 0.26 ^bc^	10.39 ± 0.18 ^c^
DPPH•, %	19.75 ± 1.35 ^ab^	20.31 ± 1.79 ^ab^	20.47 ± 1.99 ^ab^	18.01 ± 0.37 ^a^	21.72 ± 0.48 ^b^

**Table 5:** Percentage of fatty acids in black cumin seed oil, from a total fatty acid content, %. Values are means ± SD (*n* = 3). Lowercase letters “a, b, c” indicate significant differences (*p* < 0.05, Fisher’s LSD test). Treatments sharing the same letter (e.g., “a” and “b”) indicate no statistically significant difference (*p* > 0.05, Fisher’s LSD test).

Fatty Acids, %	Control	Convection Heating	Microwaves	Ultrasonication	Pulsed Electric Field (PEF)
C4:0	0.57 ± 0.01 ^b^	0.38 ± 0.12 ^a^	0.22 ± 0.02 ^a^	0.28 ± 0.12 ^a^	0.27 ± 0.13 ^a^
C6:0	0.26 ± 0.01 ^bc^	0.22 ± 0.05 ^c^	0.14 ± 0.01 ^a^	0.19 ± 0.04 ^ab^	0.16 ± 0.04 ^ab^
C8:0	0.49 ± 0.03 ^b^	0.46 ± 0.10 ^ab^	0.28 ± 0.03 ^a^	0.41 ± 0.14 ^ab^	0.37 ± 0.16 ^ab^
C10:0	0.62 ± 0.05 ^b^	0.60 ± 0.12 ^b^	0.37 ± 0.06 ^a^	0.53 ± 0.13 ^ab^	0.49 ± 0.16 ^ab^
C11:0	0	0	0	0.01 ± 0.00	0
C12:0	0	0.01 ± 0.00	0.01 ± 0.00	0.01 ± 0.00	0.01 ± 0.00
C13:0	0.90 ± 0.02 ^a^	1.05 ±0.12 ^ab^	1.15 ± 0.07 ^b^	1.13 ± 0.14 ^b^	1.13 ± 0.08 ^b^
C14:0	0.20 ± 0.01 ^a^	0.19 ± 0.01 ^a^	0.19 ± 0.01 ^a^	0.19 ± 0.01 ^a^	0.19 ± 0.01 ^a^
C14:1 cis-9	0.13 ± 0.01 ^ab^	0.18 ±0.05 ^b^	0.20 ± 0.01 ^b^	0.19 ± 0.03 ^b^	0.19 ± 0.01 ^b^
C15:0	0.04 ±0.01 ^a^	0.03 ± 0.00 ^a^	0.03 ± 0.00 ^a^	0.03 ± 0.01 ^a^	0.03 ± 0.00 ^a^
C15:1 cis-10	0.01 ± 0.00	0.01 ± 0.00	0	0.01 ± 0.00	0
C16:0	11.12 ± 0.10 ^ab^	11.48 ± 0.18 ^b^	10.73 ± 0.51 ^a^	11.31 ± 0.13 ^b^	10.99 ± 0.33 ^ab^
C16:1 cis-9	0.03 ± 0.01 ^a^	0.02 ± 0.01 ^a^	0.02 ± 0.00 ^a^	0.02 ± 0.00 ^a^	0.03 ± 0.01 ^a^
C17:0	0.06 ± 0.01 ^a^	0.06 ± 0.00 ^a^	0.06 ± 0.01 ^a^	0.07 ± 0.01 ^a^	0.06 ± 0.00 ^a^
C17:1 cis-10	0.04 ± 0.00 ^a^	0.04 ± 0.00 ^a^	0.04 ± 0.00 ^a^	0.04 ± 0.00 ^a^	0.04 ± 0.00 ^a^
C18:0	2.88 ± 0.06 ^ab^	2.91 ± 0.03 ^b^	2.79 ± 0.11 ^ab^	2.91 ± 0.04 ^b^	2.78 ± 0.08 ^a^
C18:1	23.28 ± 0.14 ^a^	23.24 ± 0.20 ^a^	23.13 ± 0.54 ^a^	23.45 ± 0.04 ^a^	23.55 ± 0.11 ^a^
C18:2	54.74 ± 0.20 ^a^	54.96 ± 0.44 ^a^	56.76 ± 0.90 ^b^	55.14 ± 0.58 ^a^	55.54 ± 1.26 ^ab^
C18:3	0.77 ± 0.09 ^b^	0.52 ± 0.09 ^a^	0.44 ± 0.05 ^a^	0.55 ± 0.10 ^a^	0.53 ± 0.20 ^a^
C20:0	0.56 ± 0.04 ^a^	0.59 ± 0.10 ^a^	0.37 ± 0.01 ^a^	0.48 ± 0.17 ^a^	0.51 ± 0.21 ^a^
C20:1	0.32 ± 0.00 ^a^	0.32 ± 0.01 ^a^	0.33 ± 0.00 ^ab^	0.34 ± 0.02 ^b^	0.34 ± 0.01 ^b^
C20:2	2.32 ± 0.01 ^a^	2.34 ± 0.02 ^ab^	2.35 ± 0.01 ^ab^	2.37 ± 0.01 ^b^	2.37 ± 0.03 ^b^
C20:3 8.11.14	0.03 ± 0.01 ^ab^	0.02 ± 0.01 ^ab^	0.02 ± 0.01 ^ab^	0.01 ± 0.00 ^a^	0.03 ± 0.01 ^b^
C20:3 11.14.17	0.01 ± 0.00 ^a^	0.01 ± 0.00 ^a^	0.01 ± 0.00 ^a^	0.01 ± 0.00 ^a^	0.01 ± 0.00 ^a^
C20:4	0.11 ± 0.1 ^c^	0.07 ± 0.02 ^b^	0.04 ± 0.01 ^ab^	0.03 ± 0.00 ^a^	0.03 ± 0.01 ^a^
C20:5	0.06 ± 0.01 ^b^	0.05 ± 0.01 ^ab^	0.03 ± 0.00 ^a^	0.02 ± 0.01 ^a^	0.03 ± 0.01 ^a^
C21:0	0.03 ± 0.00 ^a^	0.02 ± 0.01 ^a^	0.02 ± 0.01 ^a^	0.06 ± 0.05 ^a^	0.05 ± 0.04 ^a^
C22:0	0.06 ± 0.01 ^a^	0.05 ± 0.01 ^a^	0.05 ± 0.01 ^a^	0.04 ± 0.01 ^a^	0.08 ± 0.05 ^a^
C22:1	0.16 ± 0.01 ^c^	0.10 ± 0.03 ^b^	0.04 ± 0.01 ^a^	0.07 ± 0.01 ^ab^	0.07 ± 0.05 ^ab^
C22:2	0.03 ± 0.00 ^ab^	0.03 ± 0.01 ^ab^	0.02 ± 0.00 ^a^	0.02 ± 0.00 ^ab^	0.03 ± 0.01 ^b^
C22:6	0.04 ± 0.01 ^a^	0.03 ± 0.00 ^a^	0.03 ± 0.00 ^a^	0.03 ± 0.00 ^a^	0.03 ± 0.01 ^a^
C23:0	0.04 ± 0.01 ^b^	0.05 ± 0.02 ^b^	0.06 ± 0.02 ^b^	0.01 ± 0.00 ^a^	0.01 ± 0.00 ^a^
C24:0	0.02 ± 0.00 ^ab^	0.01 ± 0.00 ^a^	0.01 ± 0.00 ^abc^	0.03 ± 0.01 ^bc^	0.04 ± 0.01 ^c^
C24:1	0.06 ± 0.01 ^a^	0.06 ± 0.01 ^a^	0.07 ± 0.01 ^a^	0.06 ± 0.01 ^a^	0.06 ± 0.01 ^a^

**Table 6:** Percentage of fatty acid groups in seed oils relative to total fatty acid quantity, %. Values are means ± SD (*n* = 3). Lowercase letters “a, b, c” indicate significant differences (*p* < 0.05, Fisher’s LSD test).

Fatty Acids	Control	Convection Heating	Microwaves	Ultrasonication	Pulsed Electric Field (PEF)
SFAs	17.83 ± 0.33 ^a^	17.99 ± 0.85 ^a^	16.45 ± 0.88 ^a^	17.61 ± 1.06 ^a^	17.11 ± 1.36 ^a^
MUFAs	23.94 ± 0.16 ^a^	23.91 ± 0.28 ^a^	23.83 ± 0.56 ^a^	24.14 ± 0.11 ^a^	24.24 ± 0.16 ^a^
PUFAs	58.07 ± 0.33 ^a^	58.00 ± 0.59 ^a^	59.67 ± 0.99 ^a^	58.16 ± 0.75 ^a^	58.56 ± 1.54 ^a^
Omega-3 acids	0.10 ± 0.01 ^b^	0.08 ± 0.01 ^b^	0.06 ± 0.01 ^a^	0.05 ±0.03 ^a^	0.06 ± 0.03 ^a^
Omega-6 acids	57.98 ± 0.32 ^a^	57.92 ± 0.58 ^a^	59.61 ± 0.99 ^b^	58.11 ± 0.72 ^b^	58.50 ± 1.51 ^b^
Omega-9 acids	23.49 ± 0.16 ^a^	23.40 ± 0.24 ^a^	23.24 ± 0.55 ^a^	23.58 ± 0.11 ^a^	23.67 ± 0.17 ^a^
MUFA/PUFA	0.48 ± 0.04 ^a^	0.41 ± 0.03 ^a^	0.40 ± 0.01 ^a^	0.42 ± 0.02 ^a^	0.41 ± 0.02 ^a^
PUFA/SFA	3.26 ± 0.08 ^a^	3.22 ± 0.13 ^a^	3.63 ± 0.29 ^a^	3.30 ± 0.07 ^a^	3.42 ± 0.06 ^b^
n-6/n-3	65.06 ± 2.41 ^a^	95.38 ± 3.15 ^b^	121.00 ± 3.87 ^c^	101.90 ± 2.26 ^b^	116.38 ± 2.73 ^c^
U/S	1.34 ± 0.04 ^a^	1.33 ± 0.05 ^a^	1.45 ± 0.04 ^b^	1.37 ± 0.03 ^a^	1.42 ± 0.11 ^b^

Abbreviations in use: SFA, saturated fatty acids; MUFA, monounsaturated fatty acids; PUFA, polyunsaturated fatty acids; U/S, unsaturated fatty acid/saturated fatty acid.

**Table 7:** Tentative black cumin oil volatile aromatic compounds. Sign “+” indicates detection of the compound. Sign “−” indicates a non-detection of the compound.

Compound	Control	Convection Heating	Microwaves	Ultrasonication	Pulsed Electric Field (PEF)
(E,E)-2.4-Nonadienal	−	−	−	+	−
2-methylpropanal	+	+	+	+	+
2-propanol	+	+	+	+	+
Acetaldehyde	+	−	+	−	+
Acetophenone	−	+	−	−	−
Carvone	−	−	−	−	+
Citronellol	−	−	−	+	−
Dimethyl sulfide	+	+	+	+	+
Ethyl phenylacetate	−	−	−	+	−
Hexanoic acid	−	+	−	−	−
Nerol	−	−	+	+	−
Propanal	+	+	−	+	+

## Data Availability

The original contributions presented in this study are included in the article. Further inquiries can be directed to the corresponding author.
